# Rethinking aquatic bioindicators: testate amoebae versus metazoans in plankton samples for assessing anthropogenic impacts on freshwater ecosystems

**DOI:** 10.1007/s10661-026-15114-6

**Published:** 2026-02-27

**Authors:** João Vitor Bredariol, Matheus Henrique de Oliveira de Matos, Andressa Crystine Souza-Silva, Bianca Ramos Meira, Fábio Amodêo Lansac-Tôha, Luiz Felipe Machado Velho

**Affiliations:** Núcleo de Pesquisas em Limnologia, Ictiologia e Aquicultura (NUPELIA), Programa de Pós-graduação em Ecologia de Ambientes Aquáticos Continentais (PEA), Universidade Estadual de Maringá, Av. Colombo, 5790, CEP 87020-900 Maringá, Paraná Brazil

**Keywords:** Human pressures, National Scale Study, Rhizopoda, Amoebozoa, Zooplankton

## Abstract

Freshwater ecosystems are globally threatened, necessitating effective biomonitoring. Planktonic metazoans are key bioindicators; however, the potential of protists within the zooplankton community remains largely overlooked. We assessed the relative contribution of testate amoebae to net-zooplankton richness and density across a hydrodynamic gradient, comparing 34 lentic environments (reservoirs) with 44 lotic environments (from first- to sixth-order rivers) from a large geographic scale in Brazil. We tested the hypotheses that (i) Metazoans (rotifers, cladocerans, and copepods) would dominate in lentic systems and (ii) the contribution of testate amoebae would be greater in lotic systems, showing an inverse relationship with river order due to the exchange of fauna between compartments. The first hypothesis was confirmed, with conventional zooplankton increasing in reservoirs. The second was partially supported, while testate amoebae richness and density peaked in intermediate-order rivers, their relative contribution to the entire community was highest in lower order streams (exceeding 90%) and decreased to a maximum of 20% in larger rivers and reservoirs. Our study demonstrated that lotic ecosystems show a remarkable relative contribution of testate amoebae in plankton samples, compared with other zooplanktonic groups. Our findings emphasize the importance of incorporating protists, at least testate amoebae, into ecological assessment and river monitoring, conservation, and restoration programs. Further research is needed to elucidate how testate amoebae (and other protists) respond to different anthropogenic stressors, ideally through functional approaches and multicommunity analyses.

**Supplementary Information:**

The online version contains supplementary material available at 10.1007/s10661-026-15114-6.

## Introduction

Freshwater ecosystems, such as rivers, lakes, and ponds, cover approximately 2.3% of the Earth’s surface and harbor about 9.5% of all described animal biodiversity (Reid et al., 2019). In addition to their role in maintaining biodiversity, these systems provide essential ecosystem services for society, including the supply of vital resources such as food and water (Lynch et al., 2023). They also play a central role in human development through activities related to research, recreation, and culture (Lynch et al., 2023). However, despite their ecological and socioeconomic relevance, freshwater ecosystems are among the most threatened environments worldwide, being highly susceptible to anthropogenic pressures that drive marked environmental degradation and increase the risk of biodiversity loss (Magbanua et al., 2023).

Among the main anthropogenic impacts, those related to urbanization, agriculture, and habitat fragmentation are particularly relevant and have intensified in recent decades (Moi et al., 2022). Beyond these terrestrial pressures, one of the most detrimental human activities for freshwater biodiversity is the construction of dams for reservoir formation, which drives profound environmental transformations (Agostinho et al., 2007). These structures convert sections of lotic systems into lentic habitats, resulting in habitat loss, alterations in nutrient availability, and changes in water quality as well as in the physical and chemical properties of the system (Du et al., 2024). These alterations reduce the connectivity between water bodies and modify hydrological dynamics (Shen & Liu, 2021). All these impacts together further transform aquatic environments and directly affect biodiversity, leading to significant shifts in community structure (Guimarães Durán et al., 2024; Ticiani et al., 2023).

In this context, understanding the effects of anthropogenic pressures, particularly on fluvial systems, is of paramount importance. A key concept in this regard is the hierarchical classification of rivers, which indicates the position of a watercourse within a drainage network (Strahler, 1957). Fluvial systems of different orders exhibit distinct physical, chemical, and biological characteristics, including variations in flow velocity, depth, nutrient availability, and habitat types. These differences directly influence the composition and structure of aquatic communities along this river order gradient (Strahler, 1957; Vannote et al., 1980).

These variations in river characteristics and their influence on aquatic communities can be assessed through biomonitoring. This approach aims to detect, measure, and track environmental changes, thereby supporting management and conservation strategies in natural ecosystems (Santos & Ferreira, 2020). Within this framework, zooplankton communities are among the most widely used bioindicators of water quality (Li et al., 2019). Zooplankton collected with plankton nets exhibit high diversity in freshwater ecosystems and respond rapidly to trophic, thermal, and seasonal gradients. These organisms reflect environmental changes driven by multiple stressors, such as nutrient enrichment and the accumulation of toxic compounds (Guermazi et al., 2023; Pinto et al., 2023; Saler & Selamoglu, 2020).

Among planktonic communities, testate amoebae represent amoeboid protists characterized by the presence of a protective shell (or test) constructed by the organisms themselves (González-Miguéns et al., 2022; Silva et al., 2022). In freshwater habitats, these protists colonize a wide range of compartments, including benthonic, planktonic, and periphytic habitats in rivers, lakes, marshes, and ponds, and may occur in association with aquatic macrophytes or phytotelmata (Lansac-Tôha et al., 2014; Velho et al., 2003). However, the relevance of testate amoebae communities for biomonitoring freshwater ecosystems is often overlooked compared to other groups of the zooplankton community. This is probably because most biomonitoring studies with testate amoebae have historically focused on peatlands, swamps, and mosses, often from a palaeoecological perspective (Mitchell et al., 2008; Silva et al., 2022). Furthermore, some authors do not consider them part of the conventional zooplanktonic community, since they are protists, even though they frequently occur in zooplanktonic samples with considerable density, as demonstrated by Velho et al. (2004).

Therefore, the aim of this study was to highlight the importance of testate amoebae as a biomonitoring tool by illustrating their relative contribution in richness and density across different environments in comparison to other zooplankton groups. We examined lotic systems (first-order to sixth-order rivers) and dams (lentic environments) to understand how the testate amoebae community responds in richness and density to environmental characteristics. Specifically, we tested the following hypotheses: (i) Groups such as rotifers, copepods, and cladocerans will contribute more strongly to the richness and density of zooplankton communities in lentic environments (reservoirs), as these groups include a large number of species well adapted to true planktonic conditions. Environments with higher current velocity, such as streams and low-order rivers, are generally unfavorable for these metazoans because their low resistance to water flow limits feeding and reproduction, preventing the establishment of large populations (Aggio et al., 2022; Gomes et al., 2018). (ii) The relative contribution of testate amoebae species richness and density will be greater in lotic environments, showing an inverse relationship with river order. This pattern is expected because rivers promote the exchange of fauna from different compartments, including littoral aquatic vegetation, water column, and benthic zone, which are regions that provide suitable habitats for testate amoebae. This effect is particularly pronounced in low-order lotic environments (streams and small rivers), which are typically shallow and narrow, enhancing the contribution of testate amoebae in these habitats (Aggio et al., 2022; Alves et al., 2012; Bomfim et al., 2024; Lansac-Tôha et al., 2009; Velho et al., 2003).

## Material and methods

### Study area

**Fig. 1:**
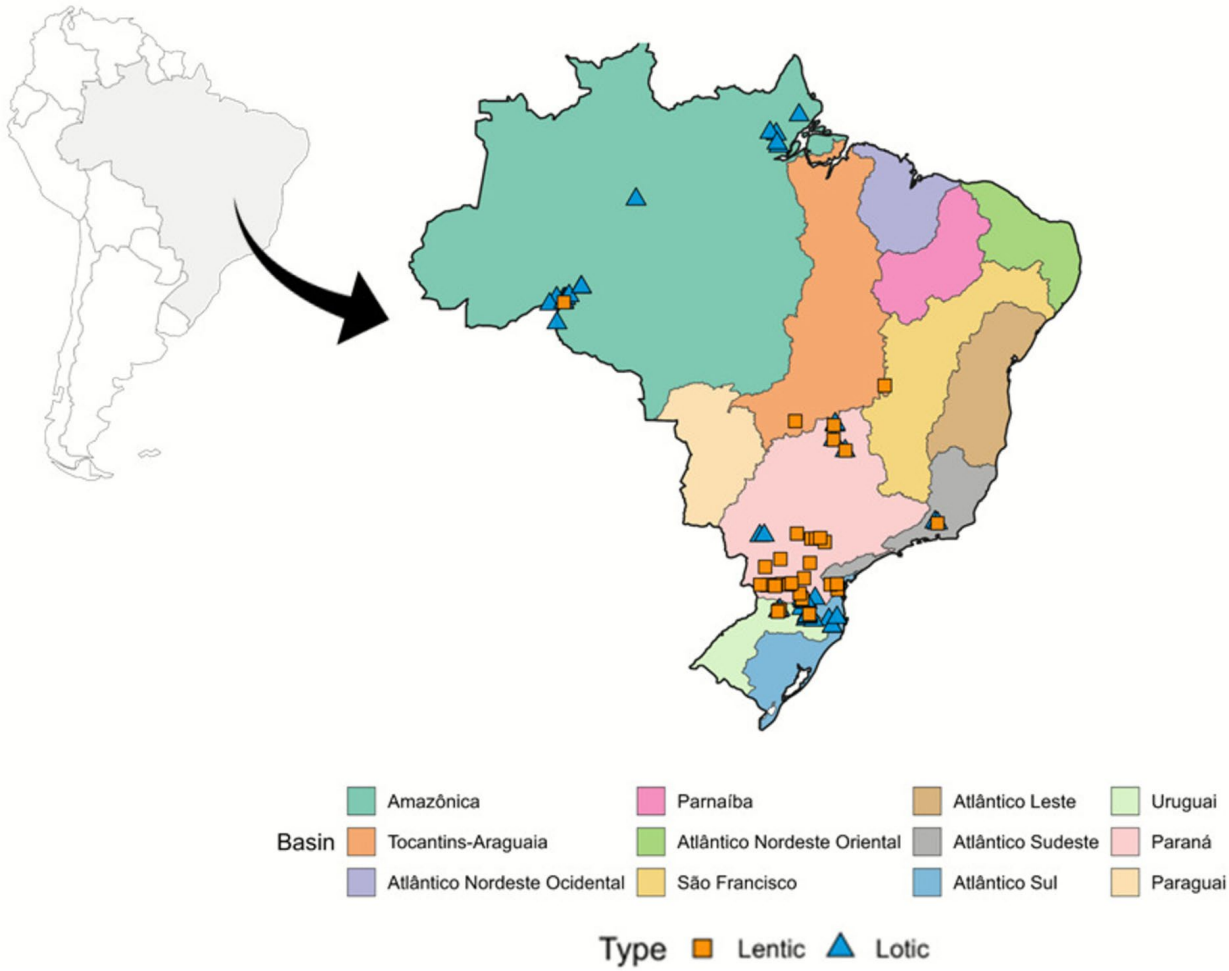
Map showing the sampling stations of lotic and lentic environments in Brazil and principal hydrographic basins in Brazil with the sampling stations

This study was performed from samples taken from 78 environments distributed throughout the Brazilian territory, including 34 lentic (reservoirs) and 44 lotic environments (Fig. 1), located in the states of Amapá, Tocantins, Amazonas, Mato Grosso, Mato Grosso do Sul, Goiás, São Paulo, Rio de Janeiro, Paraná, Santa Catarina, and Rio Grande do Sul. These environments belong to several major hydrographic basins of Brazil, including the Amazon, Tocantins-Araguaia, Paraná, São Francisco, and Southeast coastal basins, representing a wide range of hydrological, climatic, and ecological conditions. All data, collected between 2008 and 2014, were provided by biomonitoring studies in aquatic environments in Brazil.

### River order classification

The classification of river orders was carried out according to the hierarchical system proposed by Strahler (1957), which organizes lotic environments into orders based on the confluence of their tributaries. In this system, first-order streams correspond to the smallest watercourses, generally headwaters or channels without tributaries, characterized by reduced width and depth, steep slopes, and strong influence from riparian conditions, resulting in lower temperatures, high turbulence, and elevated concentrations of dissolved oxygen.

When two first-order rivers converge, they form a second-order river, which tends to exhibit a greater water volume, reduced slope, and a gradual increase in width and depth. As additional confluences occur between streams of the same order, the system evolves into intermediate-order watercourses (third–fourth order). In these systems, there is also greater heterogeneity of microhabitats and an increase in the structural and functional complexity of the aquatic community.

Fifth- to sixth-order rivers present greater hydrological connectivity, lower relative flow velocity, a predominantly finer substrate (sand, silt, and clay), higher temperatures, reduced shading from riparian vegetation, and increased environmental stability.

### Sampling and laboratory analysis

Zooplankton samples were taken at the subsurface of the central region of each environment using a pump and a plankton net (68-µm mesh size) through which from 500 to 1000 L of water was filtered. Samples were then preserved in a formaldehyde solution (4%) buffered with calcium carbonate. The samples were stained using Rose Bengal.

Among testate amoebae and other zooplankton groups, only organisms with stained protoplasm and body (indicating they were alive at the time of sampling) were counted and identified. For quantitative analyses, from each sample, three subsamples (2.5 mL each) were obtained using a Hensen–Stempell pipette and analyzed in a Sedgewick–Rafter counting chamber. At least 80 individuals were counted per sample. Samples with low organism abundance were quantified in their entirety. After counting, qualitative samples were performed, and a species accumulation curve was used to assess species richness until stabilization was reached. After counting, the abundance data was transformed to individuals per cubic meter (Ind.m^−3^) for all groups, to generate density data.

Testate amoebae, rotifers, cladocerans, and copepods were identified at the lowest taxonomic level possible, using specialized literature such as Leidy (1879), Penard (1902), Deflandre (1928), Deflandre (1929), Gauthier-Lièvre and Thomas (1958), Gauthier-Lièvre and Thomas (1960), Decloitre (1962), Vucetich (1973), Koste (1978), Segers (1995), Reid (1985), Perbiche-Neves (2011), Velho et al. (1996a, b), Elmoor-Loureiro (1997), and González-Miguéns et al. (2022). Additionally, to verify the most recent taxonomic nomenclature of taxa, online databases such as *Microworld—World of Ameboid Organisms* (Siemensma, 2025), *Tecamebas* (Gomes, 2025), *“GBIF- Global biodiversity information facility* (accessed October/2025)*”* and Cladóceros do Brasil (Elmoor-Loureiro, 2025) were consulted.

### Data analysis

To test hypotheses I and II, which aimed to evaluate the relative contribution of rotifers, copepods, cladocerans, and testate amoebae to zooplankton community richness and density across lentic and lotic environments and along the river order gradient, generalized linear models (GLMs; Nelder & Wedderburn, 1972) were employed. The Poisson family was used for testate amoebae richness, while the Gamma family was applied to density data. Comparisons across river orders also used the Gamma family for both richness and density. Model selection was based on the Akaike Information Criterion (AIC; Akaike, 1974), and assumptions of normality and homoscedasticity were verified. These approaches have been shown to reliably measure shifts in freshwater community structures.

Moreover, changes in community composition patterns across river orders were assessed using a permutational analysis of variance (PERMANOVA), based on presence–absence data (Vicente-Gonzalez & Vicente-Villardon, 2021). The analysis considered the Jaccard dissimilarity matrix with 999 permutations and a significant threshold of *p* < 0.05. When PERMANOVA results were significant, a post hoc test was performed using the “pairwise.perm.manova” function to examine differences between pairs of groups. For graphical visualization, a principal coordinates analysis (PCoA) (Gower, 1966) was performed.

All statistical analyses were performed using software R (4.4.3), coupled with R Studio (R Core Team, 2025), and utilizing the packages: *vegan* (Oksanen et al., 2022), *ggplot2* (Wickham, 2016), and *tidyverse* (Wickham et al., 2019).

## Results

### Taxonomic composition

**Fig. 2:**
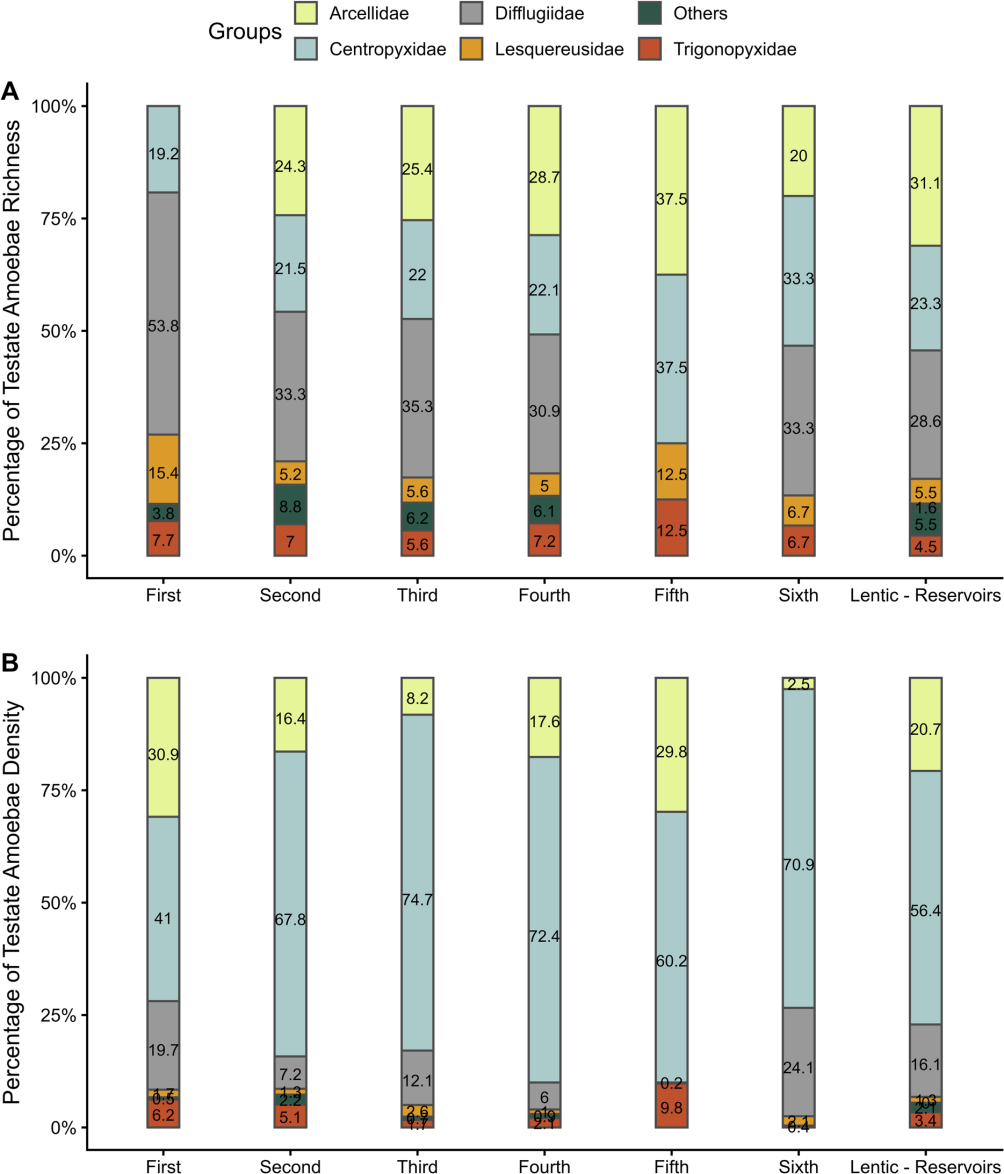
Proportion in percentage (%) of the (**A**) richness and (**B**) density of testate amoebae families among river orders and reservoirs

Throughout this study, 100 morphospecies of testate amoebae were recorded. In terms of species richness, the family Difflugiidae was generally the most dominant, except in fifth-order rivers (dominated by Centropyxidae), and reservoirs (predominance of Arcellidae) (Fig. 2). Regarding density, *Centropyxidae* was the most numerous family, particularly in third-order rivers (Fig. 2).

### Richness, density, and composition

**Fig. 3:**
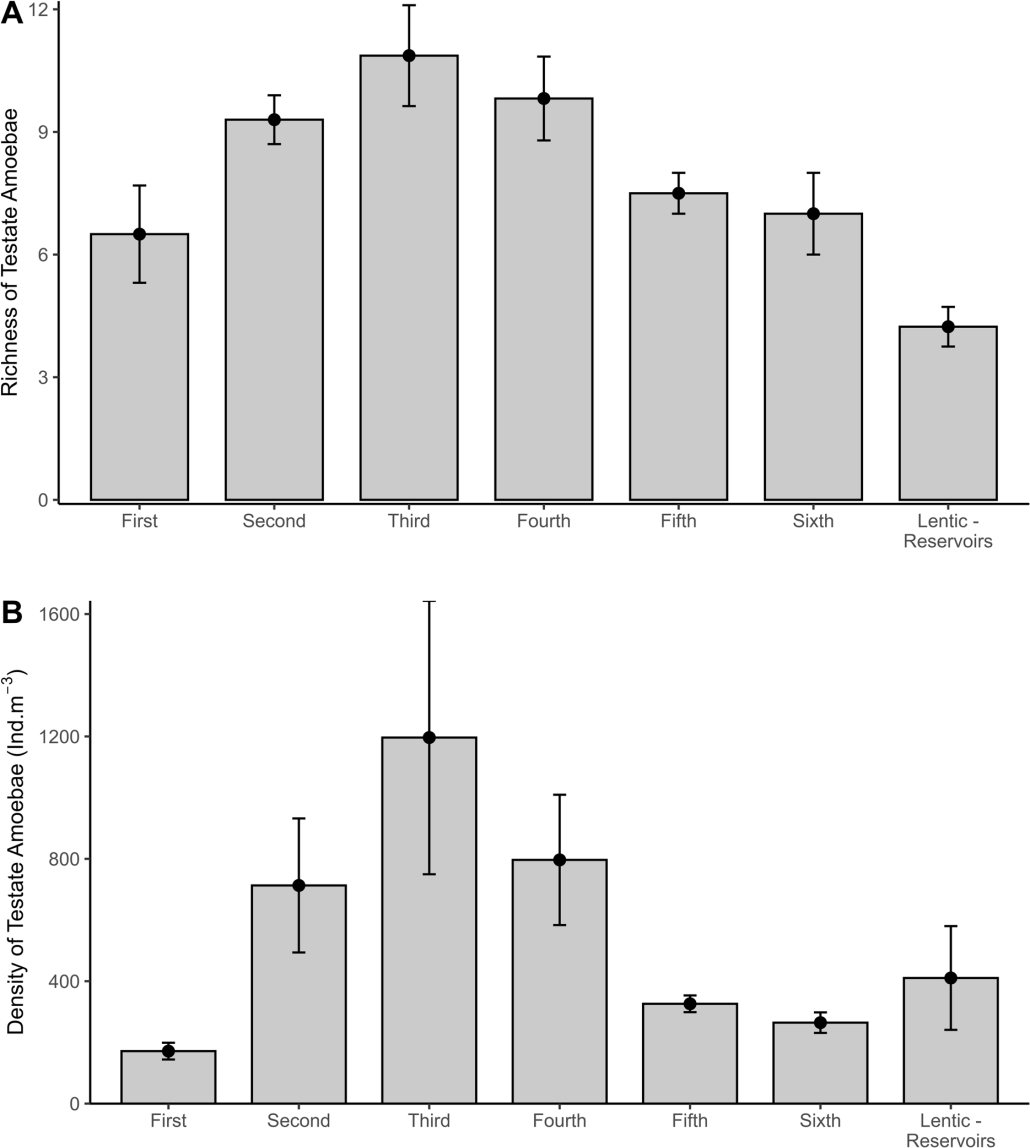
Richness (**A**) and density (**B**) of testate amoebae across river orders and reservoirs. Bars represent mean species richness (± SD) under different environments. Error bars indicate standard deviation

The richness and density of testate amoebae varied among river orders and between lotic and lentic environments. Richness was generally higher in lotic systems, particularly in intermediate-order rivers (third and fourth), whereas lentic environments (reservoirs) exhibited lower richness (Fig. 3A). Density followed a similar pattern, with lotic environments showing higher values in intermediate orders, although reservoirs displayed considerable variability for this attribute (Fig. 3B).

Generalized linear model (GLM) analyses indicated a significant effect of river order on testate amoebae richness, with strong explanatory power in the Poisson model (*R*^2^ = 0.73). Specifically, fifth-order lotic environments differed significantly from the others (Supplementary Table 1; estimated Std = 2.01; *Z* = 7.804; *p* < 0.001). Lentic environments (reservoirs) also showed a significant negative effect (Supplementary Table 1; estimated Std = −1.07; *Z* = −2.106; *p* = 0.035), indicating lower species richness under these conditions. No significant effects were observed for first-, second-, third-, fourth-, or sixth-order rivers. Regarding density, the Gamma-distributed model, with moderate explanatory power (*R*^2^ = 0.28), revealed no significant effects of lotic or lentic environment types (*p* > 0.05; Supplementary Table 1).

**Fig. 4:**
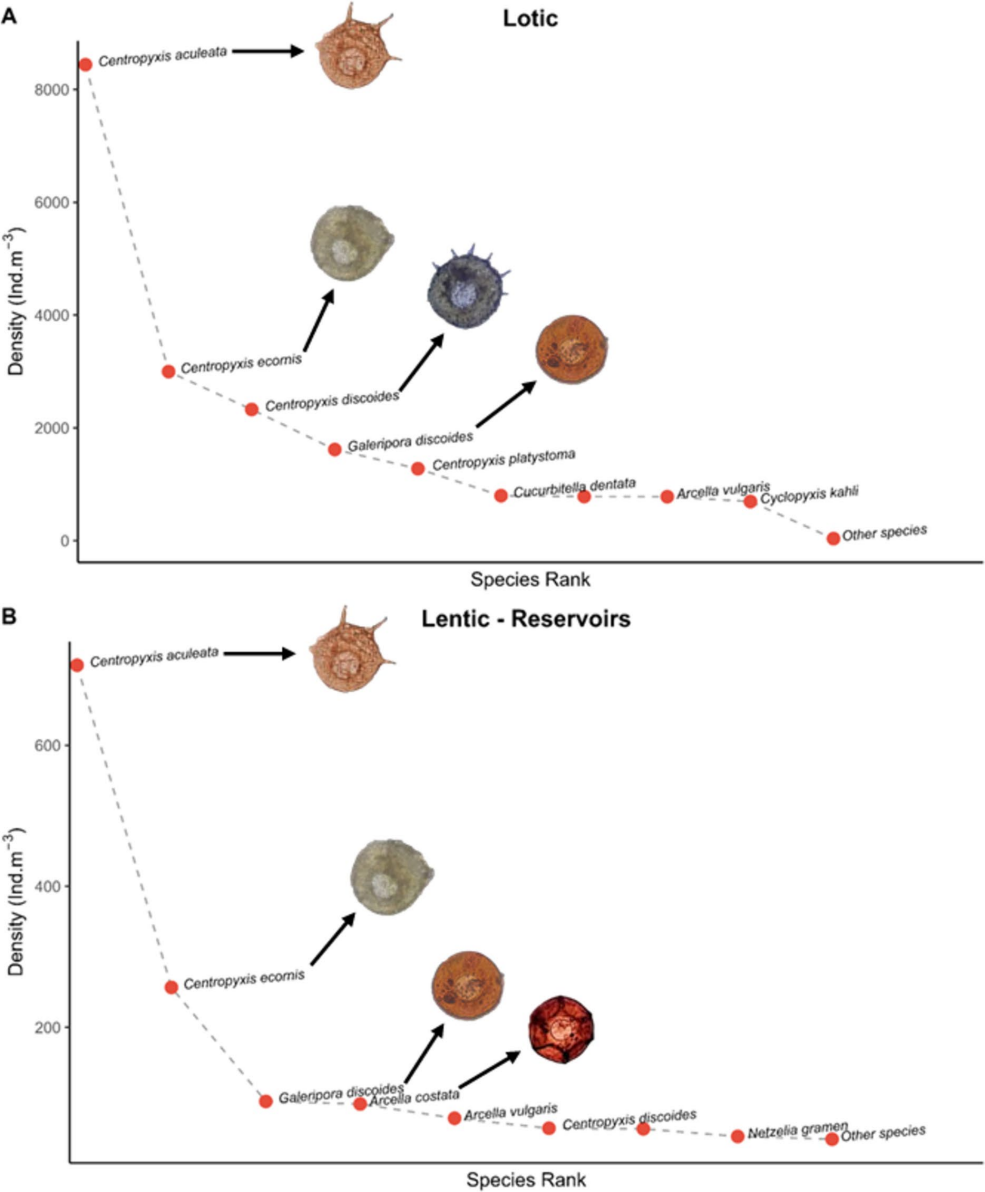
Density of testate amoebae species between lotic systems and lentic systems (reservoirs). **A** Lotic = first to sixth river order. **B** Lentic = reservoirs

*Centropyxis aculeata* (Ehrenberg, 1831) and *C. ecornis* (Ehrenberg, 1841) were the most abundant species in both lentic and lotic environments (Fig. 4), followed by *Galeripora discoides* (Ehrenberg, 1871), *Arcella costata* (Ehrenberg, 1847), and *A. vulgaris* (Ehrenberg, 1830) in lentic habitats, and *C. discoides* (Penard, 1902), *C. platystoma* (Penard, 1890), and *G. discoides* (Ehrenberg, 1871) in lotic habitats.

**Fig. 5:**
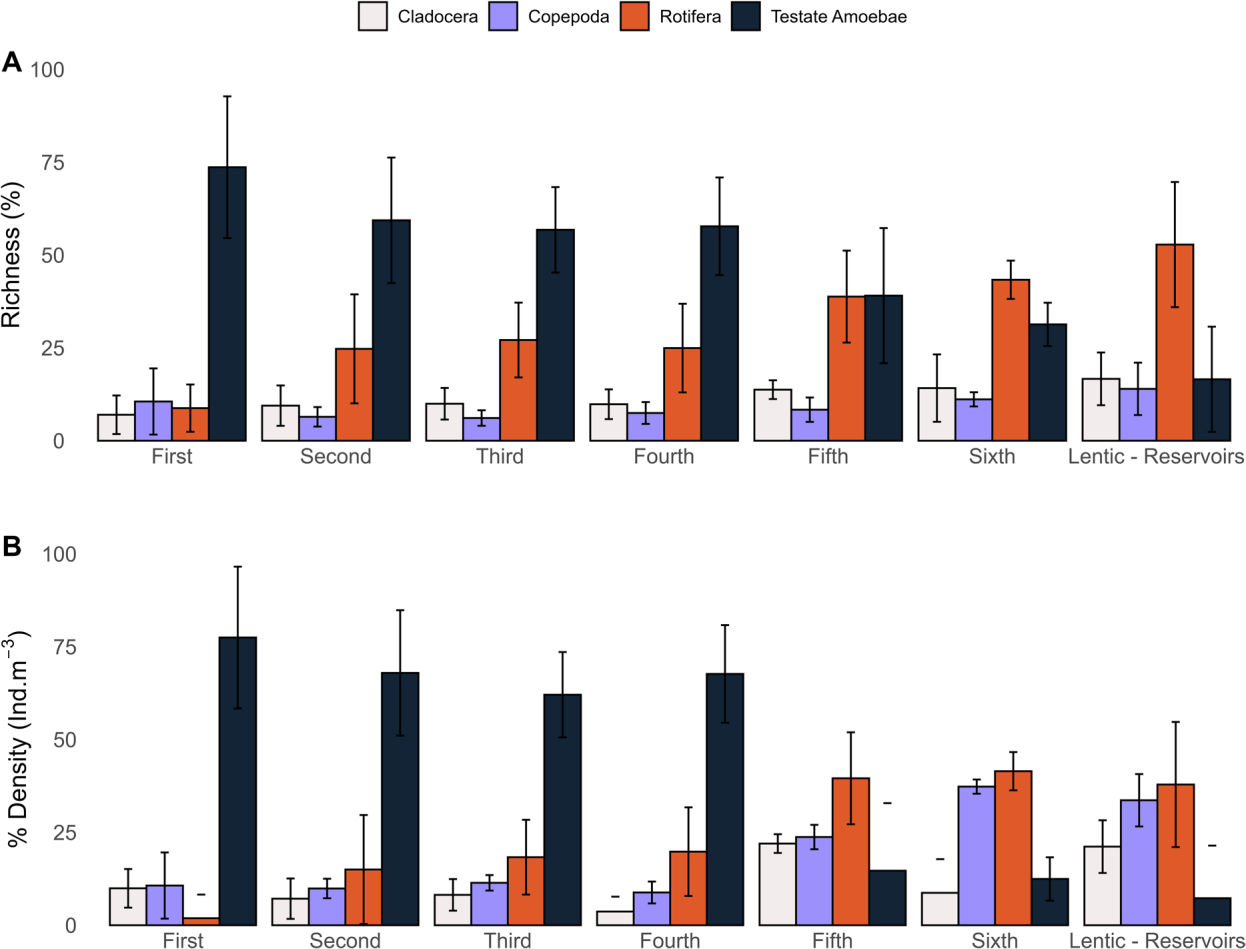
Proportion of richness (**A**) and density (**B**) of testate amoebae in relation to the zooplankton groups among river orders and reservoirs

Testate amoebae constituted the most representative group in terms of species richness and density within the zooplankton community (Fig. 5). In lotic environments, the relative importance of testate amoebae decreased markedly with increasing river order, from over 90% of the richness and density in low-order rivers to a maximum of 20% in large rivers and reservoirs.

Generalized linear models (GLMs) applied to zooplankton attributes revealed a significant interaction between river order and taxonomic groups. For species richness, assessed using Gaussian distribution, significant interactions were observed between testate amoebae and first-order (*T* = 3.106, *p* = 0.002), second-order (*T* = 2.065, *p* = 0.039), and reservoir environments (*T* = −2.299, *p* = 0.023) (Supplementary Table 2). No significant interactions were found for the other groups. Rotifera showed an isolated significant effect on species richness (*T* = 5.051, *p* < 0.001), with higher values in reservoirs.

For zooplankton density, also analyzed under a Gaussian distribution, significant interactions were detected between testate amoebae and first- (*T* = 3.100, *p* = 0.002), second- (*T* = 3.154, *p* = 0.001), third- (*T* = 2.917, *p* = 0.003), and fourth-order environments (*T* = 3.324, *p* < 0.001) (Supplementary Table 2). No significant interactions were observed for the other groups. Rotifera again exhibited an isolated significant effect (*T* = 2.839, *p* = 0.004), with increased density in fifth- and sixth-order rivers and reservoirs.

The permutational analysis of variance (PERMANOVA) revealed significant differences in testate amoebae community composition among river orders (*R*^2^ = 0.150, *F* = 2.361, *p* = 0.001). Post hoc comparisons indicated significant differences between the following pairs: first- vs. second-order (*p* = 0.038), first- vs. third-order (*p* = 0.036), second-order vs. reservoirs (*p* = 0.007), third-order vs. reservoirs (*p* = 0.007), and fourth-order vs. reservoirs (*p* = 0.007).

**Fig. 6:**
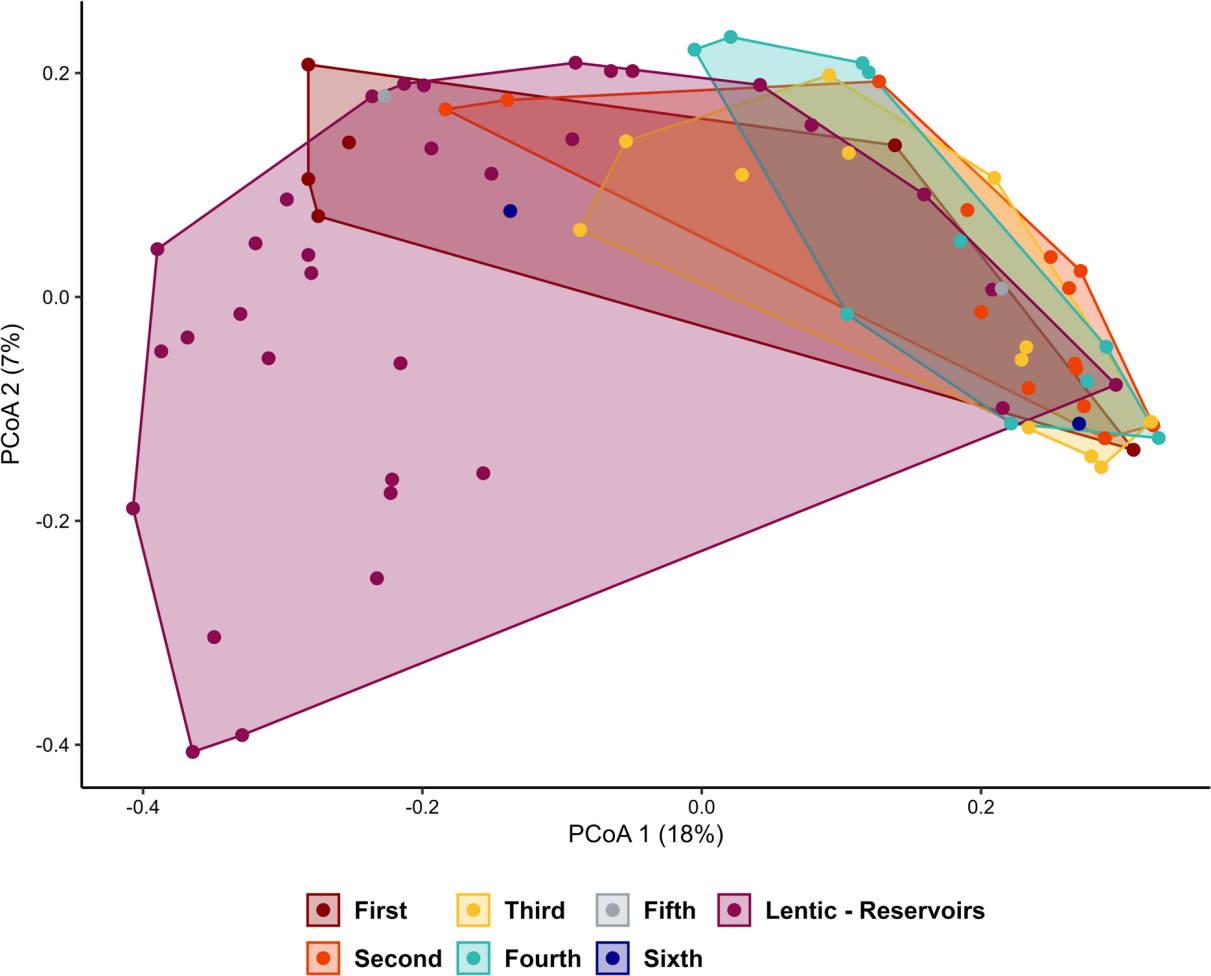
Principal coordinates analysis (PCoA) for testate amoebae species composition between river orders and reservoirs. The first axis of PCoA explained 18% of variation, and the second axis explained only 7% of variation, resulting in 25% of total variation

A principal coordinate analysis (PCoA; Fig. 6) further highlighted distinct compositional patterns among river orders. For first- to fourth-order environments, some overlap among polygons was observed, suggesting greater similarity in testate amoebae assemblages. In contrast, reservoir communities showed a broader dispersion and clear segregation from the lotic environments, indicating marked compositional differences.

## Discussion

This study highlights the importance of including the testate amoebae community as a biomonitoring tool in aquatic environments, comparing its contribution with the conventional zooplankton groups (rotifers, cladocerans, and copepods). The findings demonstrate how this community characterizes different types of river order and lentic environments with distinct physical, chemical, and biological conditions. Testate amoebae showed a strong contribution in lotic environments, particularly those subjected to intermediate hydrodynamic regimes. In contrast, lentic systems exhibited a marked decline in both diversity and density of this group. These results reinforce the importance of considering hydrological dynamics as a key variable in structuring aquatic communities and in the biomonitoring of lotic environments.

In this context, our first hypothesis was confirmed, as rotifers, cladocerans, and copepods showed a greater contribution, in terms of richness and density, than testate amoebae in lentic environments (reservoirs). Although testate amoebae were more representative in lotic environments, especially in low-order rivers, compared to other zooplankton groups, their richness and density peaked in intermediate-order rivers rather than showing a unidirectional decrease along river orders, as initially expected. This indicates that the contribution of testate amoebae is maximized under intermediate hydrodynamic conditions; therefore, rather than contradicting our second hypothesis, these results refine it by revealing a unimodal pattern of testate amoebae through the fluvial gradient, which is ecologically aligned with the expectation of the river continuum concept theory (increased habitat heterogeneity and connectivity in intermediate-order systems) (Vannote et al., 1980; see the topic “Hydrodynamics and testate amoebae diversity”).

### Structure of testate amoebae families across aquatic environments

In this study, the families Difflugiidae, Centropyxidae, and Arcellidae were the most representative in the different environments analyzed. In particular, Difflugiidae generally showed a strong contribution to species richness, whereas Centropyxidae were especially abundant in lotic environments. Arcellidae, in turn, contributed more markedly to richness in lentic environments. These families are commonly reported as dominant components of aquatic communities, both in lotic and lentic systems, as previously discussed by Velho et al. (1999), Lansac-Tôha et al. (2009), and more recently by Silva et al. (2025).

Classical studies have shown that the general morphology of these families is well adapted to aquatic habitats (Chardez, 1968). For instance, members of Difflugiidae possess shells constructed with exogenous materials from the environment, conferring high adaptability to diverse habitats and a wide range of sizes and shapes (Alves et al., 2012). This morphological variability allows this family to occupy multiple ecological niches. Velho et al. (2003) also demonstrated that environmental heterogeneity can influence testate amoebae community composition and select specific morphotypes according to habitat conditions.

For example, lotic environments with higher current velocity tend to favor discoid or flattened forms, which experience less drag (e.g., species of the genus *Centropyxis*, such as *Centropyxis aculeata* (Ehrenberg, 1838), the most representative species in the aquatic environments of this study). Inversely, more lentic environments favor oval or spherical forms with lighter, often organic shells (e.g., *Arcella* and *Galeripora*), or those capable of forming gas vacuoles, features associated with greater buoyancy and a planktonic lifestyle (Branco et al., 2023; Velho et al., 2003).

### Hydrodynamics and testate amoebae diversity

Our results demonstrated that testate amoebae are key organisms in freshwater fluvial systems, acting both as important contributors to ecosystem functioning and as bioindicators of environmental changes, such as variations in water flow velocity. When analyzing the testate amoebae community separately, we observed that third-order rivers supported the highest diversity and density of this group. These observations are consistent with the river continuum concept (RCC) proposed by Vannote et al. (1980), which highlights the importance of structural complexity and microhabitat diversity in intermediate-order streams as optimal conditions for higher biological diversity. Environments characterized by intermediate hydrodynamic regimes (transitional zones between headwaters and larger rivers), with moderate flow velocity, promote greater oxygenation, nutrient renewal, and physical heterogeneity due to the presence of a wider variety of habitats and tributary confluences (Vannote et al., 1980). Such conditions are therefore important for maintaining protist diversity, potentially supporting a greater variety of testate amoebae.

**Fig. 7:**
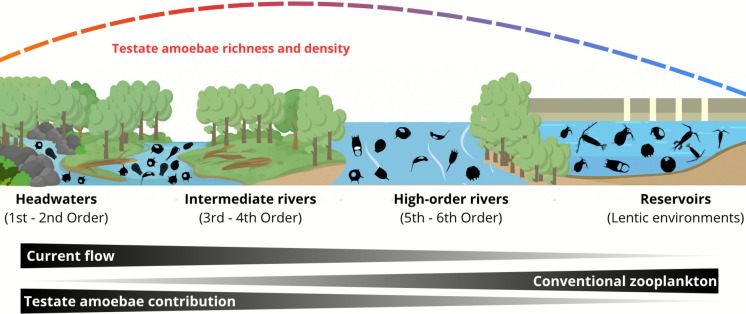
Schematic model demonstrating variations on richness and density of testate amoebae along the river order gradient, in comparison with conventional zooplankton (rotifers, cladocerans, and copepods)

Conversely, lentic environments or high-order rivers, which have larger water volumes, tend to be less heterogeneous, losing the intensity of faunal exchange among littoral, limnetic, and benthic regions. This exchange is typical of low-order lotic environments characterized by higher flow velocity, steeper slopes, and greater riparian vegetation cover (Vannote et al., 1980). Under these lentic conditions, the occurrence of testate amoebae may be limited, favoring groups better adapted to environments with lower flow velocity and greater water volume, such as conventional zooplankton (Gomes et al., 2018). Furthermore, Schwind et al. (2015) demonstrated that variations in environmental, hydrodynamic, and structural heterogeneity directly influence the composition and ecological attributes of the testate amoebae community. Our findings allowed us to propose a model that links the attributes of the testate amoebae community in comparison with conventional zooplankton, highlighting the changes in order river and disturbances caused by reservoirs (Fig. 7).

When examining the composition of aquatic communities across different river orders, we observed a higher aggregation of communities in first- to fourth-order rivers, whereas reservoirs appeared more dispersed compared to the other environments. This pattern suggests that, in lower-order rivers, zooplanktonic community composition tends to be more similar, becoming more distinct in higher order rivers and reservoirs. This process may be related to connectivity among lotic environments and dispersal processes (Aggio et al., 2022), since planktonic organisms, being suspended in the water column, can be more easily transported along the flow (Mateus-Barros et al., 2025), especially in lower order rivers with higher current velocity. Additionally, species composition may be shaped by differences in environmental heterogeneity along the fluvial gradient (Liu et al., 2025), which act as environmental filters selecting more similar species in ecologically similar sites. According to Bambakidiset al. (2024), planktonic communities from headwaters are generally compositionally more similar to nearby communities than to those located at the river mouth, due to shorter travel times, leading to more similar compositions downstream.

In the case of testate amoebae, PERMANOVA analysis showed significant differences in species composition among several river order pairs and also between lotic environments and reservoirs, indicating compositional shifts along the river order gradient. However, these compositional differences were not accompanied by changes in total density or by consistent variations in species richness across different river orders (as indicated by the GLMs), except for reservoirs which exhibited lower richness values. This may suggest that the multivariate differences detected might reflect species turnover and changes in relative abundances associated with habitat type, as also reported in the Corumbá Reservoir (Takahashi et al., 2009), rather than major shifts in overall community size throughout lotic environments.

### Bridging the gap in aquatic biomonitoring: integrating testate amoebae into lotic ecosystem assessments

In aquatic environments, the conventional zooplankton community (comprising rotifers, cladocerans, and copepods) has been widely used in ecological studies focused on water quality assessment and the detection of environmental changes, due to its trophic position and rapid response to nutrient enrichment, pollution, and habitat alterations (Goździejewska et al., 2024). However, protists, including testate amoebae, are often neglected in aquatic biomonitoring, leading to the loss of relevant information about ecosystem functioning and environmental conditions or status (Sagova-Mareckova et al., 2021).

Despite the high density of testate amoebae in zooplankton samples from various aquatic environments (Aggio et al., 2022; Arrieira et al., 2016; Lansac-Tôha et al., 2009; Silva et al., 2025; Velho et al., 2004), the potential of testate amoebae as bioindicators remains poorly understood and largely underexplored. Recently, Silva et al. (2022) conducted a comprehensive review highlighting the bioindicator potential of testate amoebae in multiple ecological contexts, emphasizing their importance in environmental monitoring due to their short life cycle, high sensitivity to environmental change, and broad distribution across aquatic and terrestrial ecosystems. Among their applications, these organisms can be employed to assess pollution, trophic status, and hydrological changes, both in modern and palaeoecological contexts, since their shells are well preserved in sediments (Mitchell et al., 2008; Regalado et al., 2018; Macumber et al., 2020; Silva et al., 2022; Rodas-Morán et al., 2022).

Thus, although testate amoebae are often an underestimated component of plankton in lotic environments, their responses to different ecological conditions allow this group to be applied across multiple scenarios, particularly where flow regimes are altered. For instance, in regulated fluvial systems, damming reorganizes the lotic–lentic continuum, intensifying longitudinal discontinuities and modifying hydrological parameters, with direct consequences for the structuring of planktonic communities and for the differentiation between upstream reaches, impounded areas, and downstream sections (Ruan et al., 2024). Within this context, the composition and abundance of testate amoebae tend to respond sensitively to these hydrological gradients, reflecting disturbance events that affect water mixing, vertical stratification, and nutrient transport (Ndayishimiye et al., 2024). Araujo et al. (2023) further emphasize that testate amoebae diversity responds to environmental pressures in streams, supporting their potential as bioindicators across drainage networks and along different impact gradients. Therefore, considering the sensitivity of testate amoebae in freshwater aquatic environments is consistent with the framework proposed by González-Miguéns et al. (2024), who classify testate amoebae as promising organisms for bioindication and monitoring, including through the use of eDNA-based protocols aimed at detecting and interpreting disturbances in continental ecosystems.

Accordingly, detectable changes in the distribution and dynamics of testate amoebae communities relative to other zooplankton groups are more clearly expressed in environments with contrasting water residence times (Ndayishimiye et al., 2025). In reaches with shorter water residence times, planktonic populations with longer life cycles (such as cladocerans and copepods) tend to experience constraints on their development, whereas organisms with higher adaptive responsiveness, rapid reproductive rates, and efficient downstream transport (i.e., protists) are favored under typical flowing-water conditions (Hromova et al., 2024). In this sense, testate amoebae, due to their representativeness under more lotic conditions and their sensitivity to hydrological gradients, may contribute to more clearly delineating the spatial compartmentalization of environments along the river–reservoir transect (Hromova et al., 2024; Ndayishimiye et al., 2024).

As demonstrated in the present study, lotic ecosystems show a remarkable relative contribution of testate amoebae in plankton samples, compared with other zooplanktonic groups. Several studies have shown that both planktonic metazoans and testate amoebae provide valuable insights in understanding ecosystem processes (Bonecker et al., 2013; Dias et al., 2016; Marcisz et al., 2016; Vieira et al., 2017; Rocha et al., 2024; Goździejewska et al., 2024). Therefore, an integrated approach combining testate amoebae with conventional zooplankton for biomonitoring in freshwater environments is crucial for a better understanding of the human impacts, such as mining, dams or land use, on ecosystem functioning. This reinforces their potential as effective bioindicators for restoration initiatives to ensure more comprehensive and accurate ecological evaluations in programs to assess freshwater ecosystems, which are currently subjected to intense anthropogenic pressures and are among the most threatened environments worldwide (Bomfim et al., 2024; Magbanua et al., 2023).

Additionally, it is also important to highlight that this study did not consider the measurements/inclusion of other important ecological drivers for testate amoebae, which may explain the low explanatory power observed in some density models. Elements such as the physicochemical variables, hydrodynamic connectivity, or temporal variability can also be important to explain changes in testate amoebae communities (Arrieira et al., 2015; Ndayishimiye et al., 2025; Schwind et al., 2016; Sysoev et al., 2024). In this context, future research should consider the integration of different drivers together with ecological data to better evaluate the ecosystem health and status.

## Conclusion

Our results revealed a notable representativeness of testate amoebae in riverine environments, particularly in lower order systems, where these organisms constituted the largest fraction of the planktonic community, when compared to conventional zooplankton. However, their diversity and density were especially pronounced in intermediate-order rivers, suggesting that the higher environmental heterogeneity presented in this type of order enhances the contribution of testate amoebae communities, especially considering the fauna exchange between compartments that happens in lower order lotic systems. In contrast, lentic environments and high-order rivers favor real planktonic groups of conventional zooplankton and limit the occurrence of testate amoebae in net-zooplankton. Hydrodynamic variation and habitat heterogeneity seem to act as primary ecological drivers for testate amoebae and overall planktonic communities in freshwater ecosystems.

Despite advances, testate amoebae remain largely overlooked in lotic ecosystem assessments compared to the long-standing use of conventional zooplankton in aquatic biomonitoring. Yet, these organisms provide valuable information about ecological structure and ecosystem functioning, particularly in environments where their heterogeneity and local hydrological fluctuations shape community dynamics. Integrating testate amoebae into biomonitoring frameworks can help bridge existing gaps in the biodiversity assessment framework and improve our understanding of how anthropogenic impacts affect freshwater ecosystems’ health. Our findings emphasize the importance of incorporating protists, at least testate amoebae, into ecological assessment and river monitoring, conservation, and restoration programs. Further research is needed to elucidate how testate amoebae (and other protists) respond to different anthropogenic stressors, ideally through functional approaches and multicommunity analyses.

## Supplementary Information

Below is the link to the electronic supplementary material.

ESM 1 Supplementary Material 1 (DOCX 49.1 KB)

## Data Availability

The datasets analyzed during the current study are not publicly available due to third-party ownership but may be obtained from the corresponding author upon reasonable request.
