# Supplementary material for: Rethinking aquatic bioindicators: testate amoebae versus metazoans in plankton samples for assessing anthropogenic impacts on freshwater ecosystems
Source: Environ Monit Assess. 2026 Feb 27;198(3):261. doi: 10.1007/s10661-026-15114-6 (PMC12948782; doi:10.1007/s10661-026-15114-6)
Supplement: Supplementary file 1 — Supplementary Material 1 (DOCX 49.1 KB) [file 10661_2026_15114_MOESM1_ESM.docx]

**SUPPLEMENTARY MATERIAL**

**Supplementary Table 1.** Generalized Linear Models (GLM) for the richness and density of testate amoebae among the river orders/environmental types. The *p*-values indicate significance at *p* < 0.05.

| **GLM for species richness** | | | | |
| --- | --- | --- | --- | --- |
|  | Estimate Std. | Error | T value | Pr(>\|t\|) |
| Intercept | 2.014 | 0.258 | 7.804 | **<0.001** |
| First order | -0.143 | 0.324 | -0.441 | 0.659 |
| Second order | 0.215 | 0.278 | 0.773 | 0.439 |
| Third order | 0.370 | 0.269 | 1.374 | 0.169 |
| Fourth order | 0.269 | 0.275 | 0.977 | 0.328 |
| Sixth order | -0.068 | 0.371 | -0.186 | 0.852 |
| Reservoirs | -0.571 | 0.271 | -2.106 | **0.035** |
| **GLM for density** | | | | |
|  | Estimate Std. | Error | T value | Pr(>\|t\|) |
| Intercept | 0.003 | 0.003 | 0.773 | 0.442 |
| First order | 0.002 | 0.006 | 0.416 | 0.679 |
| Second order | -0.001 | 0.004 | -0.411 | 0.682 |
| Third order | -0.002 | 0.003 | -0.560 | 0.577 |
| Fourth order | -0.001 | 0.004 | -0.450 | 0.654 |
| Sixth order | 0.000 | 0.000 | 0.113 | 0.910 |
| Reservoirs | -0.000 | 0.000 | -0.156 | 0.876 |

**Supplementary Table 2.** Generalized Linear Models (GLM) for the richness and density of all zooplankton groups among the river orders/environmental types. The *p*-values indicate significance at *p* < 0.05.

| **GLM for zooplankton species richness** | | | | |
| --- | --- | --- | --- | --- |
|  | Estimate Std. | Error | T value | Pr(>\|t\|) |
| Testate Amoebae | 14.686 | 7.684 | 5.086 | **<0.001** |
| Rotifera | 38.156 | 7.684 | 5.051 | **<0.001** |
| Cladocera | 13.749 | 7.684 | 1.789 | 0.074 |
| Copepoda | 8.355 | 7.684 | 1.087 | 0.277 |
| First order | -6.755 | 9.411 | -0.718 | 0.473 |
| Second order | -4.302 | 8.416 | -0.511 | 0.609 |
| Third order | -3.791 | 8.180 | -0.463 | 0.643 |
| Fourth order | -3.920 | 8.353 | -0.469 | 0.639 |
| Sixth order | 0.420 | 10.867 | 0.039 | 0.969 |
| Reservoirs | -4.302 | 8.417 | -0.511 | 0.609 |
| Copepoda : First order | 8.965 | 13.309 | 0.674 | 0.501 |
| Rotifera : First order | -23.280 | 13.309 | -1.749 | 0.081 |
| Testate Amoebae : First order | 41.336 | 13.309 | 3.106 | **0.002** |
| Copepoda : Fourth order | 3.034 | 11.813 | 0.257 | 0.797 |
| Rotifera : Fourth order | -9.941 | 11.813 | -0.842 | 0.400 |
| Testate Amoebae : Fourth order | 22.589 | 11.813 | 1.912 | 0.056 |
| Copepoda : Reservoirs | 2.700 | 11.164 | 0.242 | 0.809 |
| Rotifera: Reservoirs | 11.095 | 11.164 | 0.994 | 0.321 |
| Testate Amoebae : Reservoirs | -25.446 | 11.164 | -2.279 | **0.027** |
| Copepoda : Second order | 2.386 | 11.904 | 0.200 | 0.841 |
| Rotifera : Second order | -9.765 | 11.904 | -0.820 | 0.412 |
| Testate Amoebae : Second order | 24.587 | 11.904 | 2.065 | **0.039** |
| Copepoda : Sixth order | 2.371 | 15.386 | 0.154 | 0.877 |
| Rotifera : Sixth order | 4.114 | 15.386 | 0.268 | 0.789 |
| Testate Amoebae : Sixth order | -8.169 | 15.386 | -0.532 | 0.595 |
| Copepoda : Third order | 1.549 | 11.568 | 0.134 | 0.893 |
| Rotifera : Third order | -7.908 | 11.568 | -0.689 | 0.494 |
| Testate Amoebae : Third order | 21.520 | 11.568 | 1.860 | 0.063 |
| **GLM for zooplankton density** | | | | |
|  | Estimate Std. | Error | T value | Pr(>\|t\|) |
| Testate Amoebae | 14.686 | 13.938 | 1.054 | 0.292 |
| Rotifera | 39.571 | 13.938 | 2.839 | **0.004** |
| Cladocera | 21.991 | 13.938 | 1.578 | 0.115 |
| Copepoda | 23.750 | 13.938 | 1.704 | 0.089 |
| First order | -12.051 | 17.078 | -0.706 | 0.480 |
| Second order | -14.814 | 15.216 | -0.972 | 0.331 |
| Third order | -13.816 | 14.838 | -0.931 | 0.352 |
| Fourth order | -18.306 | 15.152 | -1.208 | 0.227 |
| Sixth order | -13.271 | 19.711 | -0.673 | 0.501 |
| Reservoirs | -0.802 | 14.320 | -0.057 | 0.950 |
| Copepoda : First order | 8.965 | 13.309 | 0.674 | 0.501 |
| Rotifera : First order | -23.280 | 13.309 | -1.749 | 0.081 |
| Testate Amoebae : First order | 41.336 | 13.309 | 3.106 | **0.002** |
| Copepoda : Fourth order | 3.034 | 11.813 | 0.257 | 0.797 |
| Rotifera : Fourth order | -9.941 | 11.813 | -0.842 | 0.400 |
| Testate Amoebae : Fourth order | 22.589 | 11.813 | 1.912 | 0.056 |
| Copepoda : Reservoirs | 2.700 | 11.164 | 0.242 | 0.809 |
| Rotifera: Reservoirs | 11.095 | 11.164 | 0.994 | 0.321 |
| Testate Amoebae : Reservoirs | -25.446 | 11.164 | -2.279 | **0.027** |
| Copepoda : Second order | 2.386 | 11.904 | 0.200 | 0.841 |
| Rotifera : Second order | -9.765 | 11.904 | -0.820 | 0.412 |
| Testate Amoebae : Second order | 24.587 | 11.904 | 2.065 | **0.039** |
| Copepoda : Sixth order | 2.371 | 15.386 | 0.154 | 0.877 |
| Rotifera : Sixth order | 4.114 | 15.386 | 0.268 | 0.789 |
| Testate Amoebae : Sixth order | -8.169 | 15.386 | -0.532 | 0.595 |
| Copepoda : Third order | 1.549 | 11.568 | 0.134 | 0.893 |
| Rotifera : Third order | -7.908 | 11.568 | -0.689 | 0.494 |
| Testate Amoebae : Third order | 21.520 | 11.568 | 1.860 | 0.063 |

**Supplementary Table 3.** List of species recorded in the study and their associated environments.

|  |  | Environment Classification (Order/Lentic) | | | | | | |
| --- | --- | --- | --- | --- | --- | --- | --- | --- |
| Family | Species | First | Second | Third | Fourth | Fifth | Sixth | Lentic - Reservoirs |
| Arcellidae | *Arcella brasiliensis* | * | * | * | * | * | * | * |
| Arcellidae | *Arcella conica* | * | * | * | * | * | * | * |
| Arcellidae | *Arcella costata* | * | * | * | * | * | * | * |
| Arcellidae | *Arcella crenulata* | * | * | * | * | * | * | * |
| Arcellidae | *Arcella gibbosa* | * | * | * | * |  | * | * |
| Arcellidae | *Arcella hemisphaerica* | * | * | * | * | * | * | * |
| Arcellidae | *Arcella mitrata* | * | * | * | * | * | * | * |
| Arcellidae | *Arcella penardi* |  | * | * |  |  | * | * |
| Arcellidae | *Arcella rotundata* |  |  |  |  |  |  | * |
| Arcellidae | *Arcella* sp. |  | * |  | * |  |  | * |
| Arcellidae | *Arcella spectabilis* | * | * | * |  | * | * |  |
| Arcellidae | *Arcella vulgaris* | * | * | * | * | * | * | * |
| Arcellidae | *Galeripora arenaria* |  | * | * | * |  |  | * |
| Arcellidae | *Galeripora artocrea* | * | * | * | * | * | * | * |
| Arcellidae | *Galeripora catinus* | * | * | * | * |  | * | * |
| Arcellidae | *Galeripora dentata* | * | * | * | * | * | * | * |
| Arcellidae | *Galeripora discoides* | * | * | * | * | * | * | * |
| Arcellidae | *Galeripora megastoma* | * | * | * | * | * | * | * |
| Arcellidae | *Galeripora rota* |  |  | * | * | * |  | * |
| Centropyxidae | *Centropyxis aculeata* | * | * | * | * | * | * | * |
| Centropyxidae | *Centropyxis aerophila* | * | * | * | * |  | * | * |
| Centropyxidae | *Centropyxis cassis* | * | * | * | * |  | * | * |
| Centropyxidae | *Centropyxis constricta* | * | * | * | * | * | * | * |
| Centropyxidae | *Centropyxis discoides* | * | * | * | * | * | * | * |
| Centropyxidae | *Centropyxis ecornis* | * | * | * | * | * | * | * |
| Centropyxidae | *Centropyxis gibba* |  | * | * | * |  | * | * |
| Centropyxidae | *Centropyxis hirsuta* | * | * | * | * |  | * | * |
| Centropyxidae | *Centropyxis marsupiformis* | * | * | * | * | * | * | * |
| Centropyxidae | *Centropyxis minuta* |  | * |  | * |  |  | * |
| Centropyxidae | *Centropyxis platystoma* | * | * | * | * | * | * | * |
| Centropyxidae | *Centropyxis* sp1 | * |  | * | * |  |  | * |
| Centropyxidae | *Centropyxis* sp2 | * |  |  |  |  |  | * |
| Centropyxidae | *Centropyxis spinosa* | * | * | * | * | * | * | * |
| Centropyxidae | *Cyclopyxis arcelloides* |  |  |  |  |  |  | * |
| Centropyxidae | *Plagiopyxis callida* | * | * | * | * |  | * | * |
| Centropyxidae | *Plagiopyxis* sp. | * | * | * | * | * | * | * |
| Cylindriflugiidae | *Cyllindrifflugia acuminata* | * | * | * | * | * | * | * |
| Cylindriflugiidae | *Cyllindrifflugia elegans* | * | * | * | * |  | * | * |
| Cylindriflugiidae | *Cyllindrifflugia lanceolata* | * | * | * | * | * | * | * |
| Cyphoderiidae | *Cyphoderia* sp. |  | * |  |  |  |  |  |
| Difflugiidae | *Difflugia amphoralis* |  |  | * |  |  |  |  |
| Difflugiidae | *Difflugia angulostoma* |  |  | * |  |  |  |  |
| Difflugiidae | *Difflugia avellana* |  |  |  |  |  |  | * |
| Difflugiidae | *Difflugia bacillifera* |  | * |  |  |  |  |  |
| Difflugiidae | *Difflugia bicruris* | * | * | * |  | * |  |  |
| Difflugiidae | *Difflugia brevicolla* |  | * | * | * |  |  | * |
| Difflugiidae | *Difflugia briophila* |  | * | * | * |  |  | * |
| Difflugiidae | *Difflugia capreolata* |  | * | * | * |  |  | * |
| Difflugiidae | *Difflugia compressa* |  | * |  | * |  |  | * |
| Difflugiidae | *Difflugia curvicaulis* |  | * | * | * |  |  |  |
| Difflugiidae | *Difflugia difficilis* |  | * | * | * |  |  |  |
| Difflugiidae | *Difflugia echinulata* | * | * | * | * |  | * | * |
| Difflugiidae | *Difflugia elongata* |  |  |  | * |  |  |  |
| Difflugiidae | *Difflugia glans* |  | * |  |  |  |  |  |
| Difflugiidae | *Difflugia globularis* |  | * | * | * |  |  |  |
| Difflugiidae | *Difflugia globulosa* |  | * | * | * |  |  | * |
| Difflugiidae | *Difflugia lebes* |  |  | * |  |  |  |  |
| Difflugiidae | *Difflugia limnetica* |  |  |  |  |  | * | * |
| Difflugiidae | *Difflugia lineare* |  | * | * | * |  | * | * |
| Difflugiidae | *Difflugia lingula* | * | * |  | * |  |  |  |
| Difflugiidae | *Difflugia lismorensis* |  | * |  |  |  |  |  |
| Difflugiidae | *Difflugia microclavicornis* | * |  |  |  |  |  |  |
| Difflugiidae | *Difflugia muriculata* | * | * | * | * |  | * |  |
| Difflugiidae | *Difflugia nebeloides* |  |  |  | * |  |  |  |
| Difflugiidae | *Difflugia oblonga* | * | * | * | * |  | * | * |
| Difflugiidae | *Difflugia parva* |  | * |  |  |  | * |  |
| Difflugiidae | *Difflugia pleustonica* | * | * | * | * |  | * | * |
| Difflugiidae | *Difflugia schurmanni* |  |  | * | * |  | * |  |
| Difflugiidae | *Difflugia* sp. | * | * | * | * | * | * | * |
| Difflugiidae | *Difflugia* sp2 |  | * | * | * |  |  |  |
| Difflugiidae | *Difflugia* sp3 |  | * | * |  |  |  |  |
| Difflugiidae | *Difflugia* sp4 |  | * | * |  |  |  |  |
| Difflugiidae | *Difflugia stellastoma* | * | * | * | * | * | * | * |
| Difflugiidae | *Difflugia urceolata* | * | * | * | * | * | * | * |
| Difflugiidae | *Difflugia ventricosa* | * |  |  |  |  |  |  |
| Euglyphidae | *Euglypha acantophora* | * | * | * | * |  |  | * |
| Euglyphidae | *Euglypha ciliata* |  |  |  |  |  |  |  |
| Euglyphidae | *Euglypha cristata* |  |  | * | * |  |  | * |
| Euglyphidae | *Euglypha filifera* | * |  | * | * |  |  |  |
| Euglyphidae | *Euglypha gauthieri* | * |  |  |  |  |  |  |
| Euglyphidae | *Euglypha rotunda* |  |  | * |  |  | * |  |
| Euglyphidae | *Euglypha* sp. | * | * |  |  |  | * |  |
| Heleoperidae | *Heleopera petricola* | * | * | * | * |  | * | * |
| Heleoperidae | *Heleopera* sp. |  |  | * |  |  | * | * |
| Hyalospheniidae | *Nebela* sp. | * | * | * | * |  | * | * |
| Hyalospheniidae | *Quadrulella* sp. |  | * |  |  |  |  |  |
| Lesquereusiidae | *Lesquereusia epistomium* |  |  | * | * |  |  | * |
| Lesquereusiidae | *Lesquereusia mimetica* |  | * | * |  |  |  | * |
| Lesquereusiidae | *Lesquereusia modesta* | * | * | * | * | * | * | * |
| Lesquereusiidae | *Lesquereusia ovalis* |  | * | * |  |  |  |  |
| Lesquereusiidae | *Lesquereusia spiralis* | * | * | * | * |  | * | * |
| Netzeliidae | *Cuccurbitella crateriformis* |  | * |  |  |  |  |  |
| Netzeliidae | *Cuccurbitella dentata* |  | * | * | * | * | * | * |
| Netzeliidae | *Cuccurbitella* sp. |  |  | * | * |  |  | * |
| Netzeliidae | *Cyclopyxis eurystoma* |  | * | * | * |  |  |  |
| Netzeliidae | *Cyclopyxis impressa* | * | * | * | * |  |  | * |
| Netzeliidae | *Cyclopyxis kahli* | * | * | * | * | * | * | * |
| Netzeliidae | *Cyclopyxis* sp. | * | * | * | * |  | * | * |
| Netzeliidae | *Netzelia corona* | * | * | * | * | * | * | * |
| Netzeliidae | *Netzelia gramen* | * | * | * | * |  | * | * |
| Netzeliidae | *Netzelia labeosa* |  |  | * | * |  |  | * |
| Netzeliidae | *Netzelia lithophila* | * | * | * | * | * | * | * |
| Netzeliidae | *Netzelia lobostoma* | * | * | * | * | * | * | * |
| Netzeliidae | *Netzelia muriformis* | * | * | * | * |  |  | * |
| Netzeliidae | *Netzelia oviormis* |  | * | * | * |  |  | * |
| Netzeliidae | *Netzelia tuberculata* | * |  | * | * |  | * | * |
| Netzeliidae | *Netzelia wailesi* |  |  | * |  |  |  |  |
| Phryganellidae | *Phryganella dissimulatoris* |  | * | * |  |  |  |  |
| Phryganellidae | *Phryganellla* sp. | * | * | * |  |  |  | * |
| Trinematidae | *Trinema enchelys* |  |  | * | * |  | * |  |
| Trinematidae | *Trinema lineare* |  |  | * |  |  |  |  |
| Trinematidae | *Trinema sp.* |  |  | * |  |  |  |  |
| Incertae sedis | *Hoogenraadia sp.* |  |  |  |  |  | * |  |
| Incertae sedis | *Hoogenraadia cryptostoma* |  | * | * | * | * | * | * |
| Incertae sedis | *Lagenodifflugia vas* |  | * |  |  |  |  |  |
| Incertae sedis | *Pentagonia marrocana* |  | * |  |  |  |  |  |
| Incertae sedis | *Pontigulasia compressa* | * | * | * | * |  | * | * |
| Incertae sedis | *Pontigulasia* sp. | * | * | * | * |  | * | * |
| Incertae sedis | *Protocuccurbitella coroniformis* | * | * | * | * |  |  | * |
| Incertae sedis | *Trigonopyxis arcula* | * | * | * | * |  | * | * |
